# Supplementary material for: Coexistence of Genetic Diseases Is a New Clinical Challenge: Three Unrelated Cases of Dual Diagnosis
Source: Genes (Basel). 2023 Feb 14;14(2):484. doi: 10.3390/genes14020484 (PMC9957527; doi:10.3390/genes14020484)
Supplement: Supplementary file 1 [file genes-14-00484-s001.zip › genes-2191314-supplementary.pdf]

Table S1. Genetic conditions and phenotypes correlations.

	GENETIC CONDITIONS	
**CASE 1**	10q11.22q11.23 microduplication	Variants in *WDR19* gene.<br>NM_025132.3: c.[3470A>G];[3470A>G]
**CASE 2**	47,XY+21	Variants in *LAMA2* gene.<br>NM_000426.3: c.[850G>A];[5374G>T]
**CASE 3**	16p11.2 microdeletion	Variants in *ABCA4* gene.<br>NM_000350: c.[2828G>A];[2828G>A]

**PHENOTYPE CASE 1**	dup10q11.22q11.23 certanly associated	dup10q11.22q11.23 posibily associated	*WDR19* gene certanly associated	*WDR19* gene posibily associated
trigonocephaly				yes
microcephaly		yes		
short stature		yes		
toe and finger cutaneous syndactyly		yes		
dysplasia of the aortic valve		yes		
intellectual disability		yes		
myopia		yes		
chronic kidney desease				yes
polycystic kidney				yes
polycystic liver disease				yes

**PHENOTYPE CASE 2**	47,XY+21 certanly associated	47,XY+21, posibily associated	*LAMA2* gene certanly associated	*LAMA2* gene posibily associated
by type A ventricular atrium canal	yes			
criptorchydism	yes			
severe hypotonia	yes		yes	
distal muscle weakness			yes	
delayed psychomotor development			yes	
elevated CPK			yes	

**PHENOTYPE CASE 3**	del16p11.2 certanly associated	del16p11.2 posibily associated	*ABCA4* gene certanly associated	*ABCA4* gene posibily associated
obesity	yes			
epilepsy	yes			
myopia	yes			
hypogonadism	yes			
scoliosis	yes			
tapering fingers	yes			
insufficiency of mitral tricuspid and aortic valves	yes			
delayed psychomotor development	yes			
mild intellectual disability	yes			
autism	yes			
Stargardt maculopathy			yes	
